# Biochemical characterization of the fidelity of poliovirus RNA-dependent RNA polymerase

**DOI:** 10.1186/1743-422X-4-44

**Published:** 2007-05-24

**Authors:** Marion S Freistadt, Joseph A Vaccaro, Karen E Eberle

**Affiliations:** 1 Department of Microbiology, Immunology and Parasitology; Louisiana State University Health Sciences Center, 1901 Perdido St., New Orleans, Louisiana, 70112, USA; 2 Department of Cell and Molecular Biology, 2000 Stern Hall, 6400 Freret St, Tulane University, New Orleans, LA, 70118, USA; 3 Tulane University Health Sciences Center, Department of Biochemistry,1430 Tulane Avenue SL-43, New Orleans, LA 70112-2699, USA

## Abstract

**Background:**

Putative high mutation rates of RNA viruses are believed to mediate undesirable phenomena, such as emergence of drug resistance. However, very little is known about biochemical fidelity rates for viral RNA-dependent RNA polymerases. Using a recently developed *in vitro *polymerase assay for poliovirus polymerase 3D^pol ^[Arnold and Cameron (2000) JBC 275:5329], we measured fidelity for each possible mismatch. Polymerase fidelity, in contrast to sequence error rate, is biochemically defined as *k*_*pol*_/*K*_*d *_of {(correct plus incorrect) divided by incorrect} incorporations, such that a larger value connotes higher fidelity.

**Results:**

To derive *k*_*pol*_/*K*_*d *_for correct base incorporation, we performed conventional pre-steady state single turnover measurements, yielding values that range from 0.62 to 9.4 μM^-1 ^sec^-1^. Pre-steady state measurements for incorrect base incorporation were less straightforward: several anomalous phenomena interfered with data collection. To obtain pre-steady state kinetic data for incorrect base incorporation, three strategies were employed. (1) For some incorrect bases, a conventional approach was feasible, although care was taken to ensure that only single turnovers were being assessed. (2) Heparin or unlabeled RNA traps were used to simulate single turnover conditions. (3) Finally, for some incorrect bases, incorporation was so poor that single datapoints were used to provide kinetic estimates. Overall, we found that fidelity for poliovirus polymerase 3D^pol ^ranges from 1.2 × 10^4 ^to 1.0 × 10^6 ^for transition mutations and 3.2 × 10^5 ^to 4.3 × 10^7 ^for transversion mutations.

**Conclusion:**

These values are unexpectedly high showing that high RNA virus sequence variation is not due to intrinsically low polymerase fidelity. Based on unusual enzyme behavior that we observed, we speculate that RNA mismatches either directly or indirectly cause enzyme RNA dissociation. If so, high sequence variation of RNA viruses may be due to template-switch RNA recombination and/or unknown fitness/selection phenomena. These findings may lead to a mechanistic understanding of RNA virus error catastrophe and improved anti-viral strategies.

## Background

RNA viruses are responsible annually for worldwide morbidity and mortality of millions of people, animals and plants. Key features that render RNA viral diseases particularly intractable are rapid emergence of drug resistance, vaccine escape and emergence of novel pathogens. These undesirable properties of RNA viruses are thought to be mediated by "quasi-species" phenomena. Recent theoretical and experimental work render quasi-species a useful framework by which we may understand RNA viruses [1]. In this model, an RNA virus population exists at its highest possible ("threshold") mutation rate, maximizing adaptability while ensuring population survival. This effect is manifested as extreme genetic heterogeneity, measured by sequence variation [2]. A number of RNA viruses appear to have relatively high sensitivity to mutagens, supporting the idea that RNA viruses exist at a threshold mutation rate [3-9]. This renders "lethal mutagenesis" a potential novel anti-viral strategy [10].

The RNA-dependent RNA polymerases (RdRPs) lack of exonucleolytic editing is often cited as the reason that RNA viruses have high sequence variation [1]. If so, one would expect sequence variation rates to resemble RdRP fidelity values. A review of the literature reveals that there are few biochemical studies of RdRP fidelity; studies that exist suggest a lack of quantitative correlation between polymerase fidelity and sequence variation [5,11,12].

Poliovirus (PV), one of the best understood and experimentally amenable viral model systems, is a member of the picornavirus family of RNA viruses. Replication of the RNA genome is catalyzed by the viral RdRP, 3D^pol^. The structure of 3D^pol ^has been solved at 2.0 Å resolution and, like other DNA and RNA polymerases, resembles a right hand with fingers, palm, and thumb domains [13,14] {pdb: 1RDR and 1RA6}. Recently, difficulties in assaying this enzyme have been overcome by the development of expression and assay systems that permit the assembly of stoichiometric enzyme primer/template complexes and subsequent evaluation of specific nucleotide incorporation products by end-labeled primer extension analysis [15]. Earlier work measuring 3D^pol ^error rates used steady state conditions and heterogeneous template/products, precluding gel product analysis [16]. These advances have led to a detailed transient-state kinetic analysis of RNA-dependent RNA polymerization by 3D^pol ^[11,17]. These studies have shown that the reaction pathway for the enzyme first involves the slow binding of the primer/template to 3D^pol ^followed by a conformational change that produces a stable, catalytically competent binary complex. An incoming nucleotide can then bind to form a catalytically inactive ternary complex that must undergo a conformational change in order for chemistry to occur. Following the phosphoryl transfer step, a third isomerization occurs that allows the dissociation of inorganic pyrophosphate from the ternary product complex. The resulting 3D^pol ^extended-primer/template product complex is catalytically ready for the next round of nucleotide binding and incorporation.

The development of this model for RNA-dependent RNA polymerization by 3D^pol ^has permitted the current study of this enzyme's fidelity. Our long-term goal is to understand the structural basis of RdRP fidelity, its role in RNA virus pathogenesis and to use this information to improve anti-virals. Arnold and Cameron [17] performed pre-steady state analysis for one incorrect base, (GTP templated by U). In the current study, we employed their *in vitro *system to measure 3D^pol ^intrinsic fidelity. Here we present the first pre-steady state biochemical fidelity measurements for poliovirus polymerase 3D^pol ^for all twelve possible mismatches. We expected 3D^pol ^to behave similarly to HIV RT with measurably low fidelity. Instead, we found surprisingly large differences between correct and incorrect nucleoside incorporation.

## Results

### 1. Pre-steady state rate measurements for correct nucleotides

**Figure 1:**
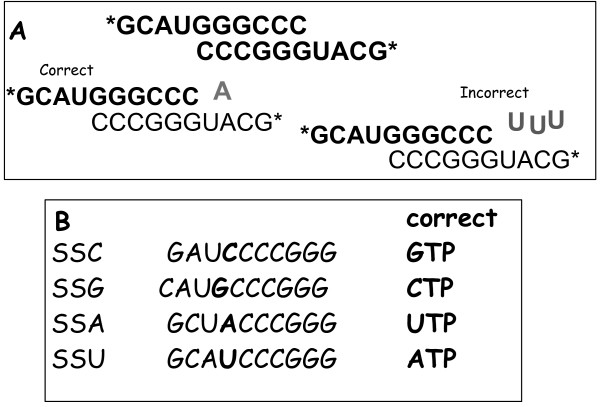
**RNA substrates used**. (A) Sequence of "Symmetrical/Substrate U". Commercially synthesized RNA 10 mers were prepared and phosphorylated with isotopic [γ^32^P] label (*) as described in Methods. After melting and annealing, the RNA molecules self-anneal to form a six base-pair duplex with identical 5' overhangs. For Sym/Sub U, the correct base at N + 1 position, A, and one incorrect base, U, is shown (incorporated bases shown in lighter type). (B) List of the four Sym/Subs used in this study. Four similar synthetic RNAs were used. The first template base is shown in bold, and the correct base indicated in the right-hand column.

To determine polymerase fidelity, pre-steady state analyses to establish *k*_*pol *_and *K*_*d *_for correct and incorrect bases are required [18]. To do this for 3D^pol^, we used a set of four, end-labeled, symmetrical substrate RNAs ("Sym/Sub" or "SS") (Fig. 1) [15]. Each 10-base Sym/Sub is self-annealing and functions, after annealing, as primer/template. The Sym/Subs used in this study differ from each other only in the order of the four overhang template bases; each is named for the first template base, e.g., Sym/Sub U has uridine as the first template base and is correctly matched with adenosine (Fig. 1). By varying Sym/Sub or NTP, each base may be used as template, with each correct or incorrect substrate, permitting biochemical assessment of all 16 possible basepair combinations. Note that, due to the self-annealing property of Sym/Subs, each duplex has two labeled ends, such that the maximum possible incorporation in a single turnover is 50% of input labeled ends.

**Figure 2:**
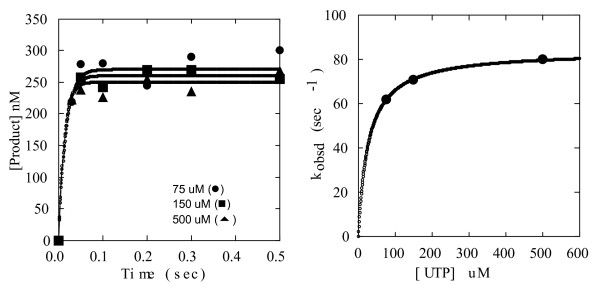
**Single turnover analysis (rapid quench) of UTP (correct) incorporation into SSA**. (A) Product yield as a function of time for three concentrations of UTP. A series of single turnover, rapid quench reactions were performed and analyzed described in Methods. Product concentration of resulting 11 mer (in nM) as a function of time in seconds is shown for 75 (circles), 150 (squares) and 500 (triangles) μM UTP. Other reagents' concentrations were: 3D^pol^: 1 μM, Sym/Sub A: 1 μM (= 0.5 μM duplex). Data from each UTP concentration were fit to a single exponential. Amplitudes and observed reaction rates are given in Table 1. (B) Observed reaction rates as a function of UTP concentration. Data were fit to a hyperbola. *K*_*d *_and *k*_*pol *_are given in Table 1.

For each correct incorporation reaction, a pre-steady state kinetic analysis was conducted under single turnover, rapid quench conditions, in which enzyme was in slight excess of RNA substrate. Confirmation of enzyme excess was determined as described in Methods. In correct reactions, only 11 mers were produced. Quantified product yield at each timepoint (7–10 points under 1 second) was used to derive amplitude and observed reaction rate (*k*_obsd_) by fitting data to a single exponential. (shown for Sym/Sub A with UTP in Fig 2A). A series of these pre-steady reactions were then performed at various NTP concentrations (Table 1). With increasing NTP concentration, observed reaction rates increased. Observed reaction rates were then plotted as a function of NTP concentration and data fit to a hyperbola to derive *K*_*d *_and *k*_*pol *_(Fig. 2B and Table 1). The values obtained for incorporation of UMP into Sym/Sub A appeared similar to published values for 3D^pol^. We performed correct incorporation measurements for the four correct reactions as controls for incorrect measurements. However, detailed experiments were not pursued since these values have already been reported. [15,19].

**Table 1:** 

**Sym-Sub U**		
**[ATP] (μM)**	**Amplitude (nM)**	**kobs (sec-1)**
50	110 (± 4.4)	24 (± 4.7)
100	160 (± 25)	30 (± 21)
200	100 (± 6.1)	47 (± 16)
***K***_*d *_**(**μ**M)**	***k***_*pol *_**(sec-1)**	**kpol/Kd (**μ**M**^-1 ^**sec**^-1^**)**
120 (± 74)	74 (± 33)	0.62
**Sym-Sub C**		
**[GTP] (**μ**M)**	**Amplitude (nM)**	**kobs (sec-1)**
1	150 (± 13)	6.7 (± 1.1)
2	120 (± 4.7)	9.7 (± 1.3)
3	120 (± 13)	9.9 (± 3.4)
***K***_***d ***_**(**μ**M)**	***k***_*pol *_**(sec-1)**	**k**_**pol**_**/*Kd *(**μ**M**^-1 ^**sec**^-1^**)**
0.93 (± 0.45)	13 (± 2.0)	9.4
**Sym-Sub A**		
**[UTP] (**μ**M)**	**Amplitude (nM)**	**kobs (sec-1)**
75	270 (± 13)	62 (± 17)
150	260 (± 6.7)	71 (± 25)
500	250 (± 71)	80 (± 21)
***K***_***d ***_**(**μ**M)**	***k***_***pol ***_**(sec-1)**	**k**_***pol***_**/*Kd *(**μ**M**^-1 ^**sec**^-1^**)**
27 (± 0.95)	84 (± 0.48)	3.1
**Sym-Sub G**		
**[CTP] (**μ**M)**	**Amplitude (nM)**	**kobs (sec-1)**
10	89 (± 16)	10 (± 4.8)
25	100 (± 36)	19 (± 2.1)
100	110 (± 38)	38 (± 5.8)
***K*_***d ***_(**μ**M)**	***k*_***pol ***_(sec-1)**	**k_**pol**_/*Kd *(**μ**M**^-1 ^**sec^-1^)**
44 (± 7.2)	55 (± 3.8)	1.25

### 2. Pre-steady state rate measurements for incorrect nucleotides: inhibition by high NTP concentration

To derive pre-steady state kinetic values for incorrect nucleotides, similar experiments were performed with the four templates, except, in each case, a single incorrect base (for the first template base) was provided. Since by definition, incorrect base incorporation is much less efficient than correct, we expected less efficient NTP binding and slower reaction rates for incorrect nucleotides. Therefore higher NTP concentrations and longer reaction times were employed, while maintaining enzyme excess.

**Figure 3:**
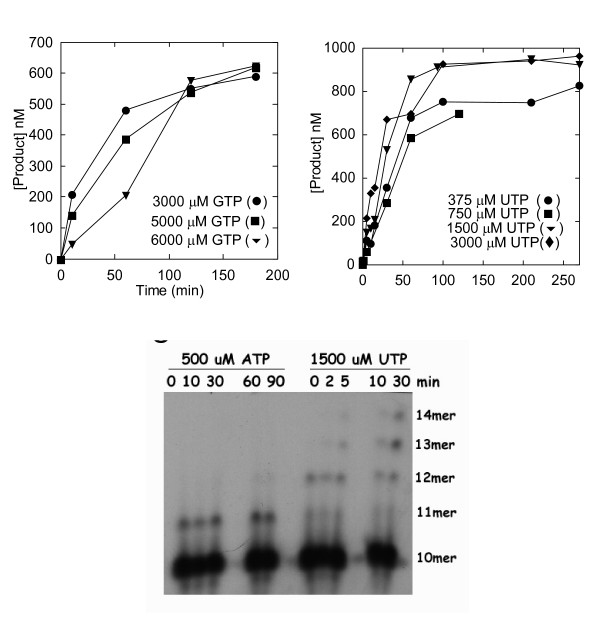
**Incorrect incorporation into Sym/Sub U**. (A) Apparent inhibition by high NTP concentrations. Products formed in the presence of 3000 (circles), 5000 (squares) and 6000 (triangles) μM GTP were quantified and plotted as a function of time in seconds. All products were included in the calculations for this graph. These data did not fit a single exponential: the line is provided only for improved viewing. (B) Apparent multiple turnovers with incorrect base. Quantified products (all) formed in the presence of 375 (circles), 700 (squares), 1500 (triangles) and 3000 (diamonds) μM UTP and plotted as a function of time in seconds. These data did not fit a single exponential: the line is provided only for improved viewing. (C) Formation of higher molecular weight products in the presence of incorrect, but not, correct NTP. Left five lanes: 1 μM Sym/Sub U incubated with 500 μM ATP (correct base) for the indicated times (in minutes). Right five lanes: 1 μM SSU incubated with 1500 μM UTP (incorrect base) for indicated time (minutes). Samples were denatured and electrophoresed in a 20% denaturing PAGE. Size of RNA species is indicated at the right of the gel. 10 mer is the starting material.

During these experiments, several unexpected phenomena were noted. First, we found that 3D^pol ^was inhibited by NTP concentrations higher than 2 mM (Fig. 3A). Although Fig. 3A shows Sym/Sub U with G, similar phenomena were noted with other template/substrate combinations. Increased magnesium did not ablate this effect (data not shown). Therefore, lower NTP concentrations were necessitated; however, this often precluded approaching presumptive *K*_*d *_values. Moreover, the relatively low NTP concentrations yielded relatively inefficient incorporation.

### 3. Apparent multiple turnovers for incorrect bases

Taking these constraints under consideration, we observed an apparently paradoxical phenomenon: greater than 500 nM product yield. Under the conditions employed, 100% incorporation of a single nucleotide in a single turnover should yield 500 nM product (the starting concentration of duplexed template/primer; but 50% of input labeled ends). However, we noted greater than 500 nM product with most Sym/Sub and incorrect base combinations (Fig. 3B). This should not happen when enzyme is in excess, as it was here. Greater than 500 nM incorporation is possible by extension or dissociation, re-association and incorporation at the second free 3' end of the annealed Sym/Sub (Fig. 1). The input labeled Sym/Sub concentration, 1000 nM, was the maximum possible, allowing full reuse of template/primer. To compare the effect of nucleotide correctness on length of product, we incubated SSU with correct or incorrect base for extended times. We observed different effects: after incorporating a correct base, the complex was stable and no further incorporation was observed: this is consistent with early work on 3D^pol ^showing extremely high complex stability [15,20]. However, after incorporating an incorrect base, multiple catalytic rounds occurred, generating 11-, 12-, 13- and 14-mers (Fig. 3C) in the presence of incorrect NTP. No untemplated synthesis was observed.

These difficulties made derivation of accurate pre-steady state kinetic values for incorrect incorporation challenging. Therefore, we employed several approaches to overcome this. First, we attempted to estimate kinetic values by estimating the time at which the first turnover ended, assuming synchronicity. Therefore, in these inefficient, incorrect reactions, product incorporation greater than theoretical 100% (500 nM or 50% input labeled ends) was disregarded during derivation of kinetic values (Table 2). One additional aberration was observed in reactions consisting of Sym/Sub U with UTP: only 12- and 13 mers (no 11 mers) were detected. Presumably this is due to the relatively rapid incorporation of UMP templated by A at the N+2 position (Fig. 1); however, this phenomenon was not observed in the other similar cases. For these reactions only, data from 12 mers was used to approximate 11 mer data, since the quantitative contribution of the correct reaction to the incorrect reaction is very likely to be negligible.

**Table 2:** 

**NTP**	***Kd *(uM)**	Factor*	**k_**pol **_(sec-1)**	Factor*
ATP	1.2 (± 0.74) × 10^2^		74 (± 22)	
UTP-untrapped**	1.7 (± 0.53) × 10^5^		0.14 (± 0.052)	
UTP-heparin trap***	5.3 (± 3.0) × 10^4^	-3.2	0.032 (± 0.02)	-4.4
UTP-cold trap***	5.3 (± 0.065) × 10^4^	-3.2	0.027 (± 4.3 × 10^-4^)	-5.2

*Factor by which data with trap differ from data without trap.

**Only 12 mers were analyzed (because no 11 mers were visible). Data over 500 nM were disregarded.

***All data.

### 4. Traps to measure pre-steady state rate for incorrect base incorporation

**Figure 4:**
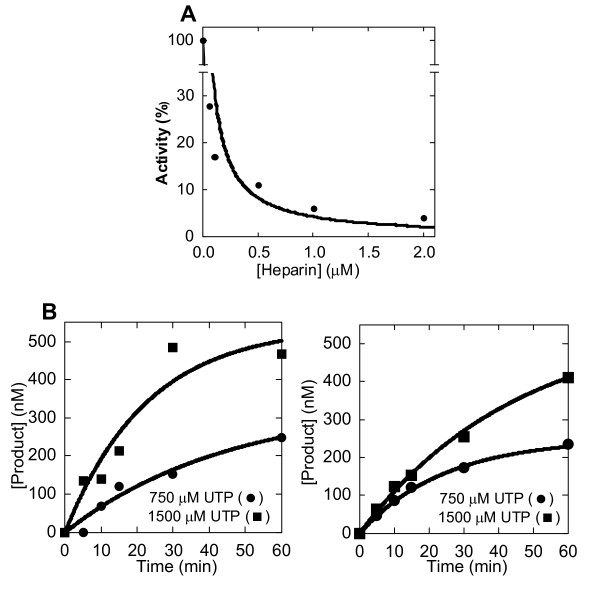
**Traps to reduce multiple turnovers**. (A) Titration of heparin inhibition of 3D^pol^. 1 μM Sym/Sub U was incubated with 500 μM ATP, 1 μM 3D^pol ^and increasing amounts of heparin. Polymerase activity was assessed in 20% PAGE, as described in Methods. The data were fit to a single exponential curve for inhibition. (B) Apparent single turnover kinetics for 3D^pol ^with Sym/Sub U and incorrect base U in the presence heparin trap. 1 μM 3D^pol ^and 1 μM Sym/Sub U were incubated with 750 (squares) or 1500 μM UTP (circles) for the indicated time (in minutes) as described in Methods, except that 100 nM heparin was added immediately after reaction initiation. Data (from all products) were plotted as a function of time in seconds and fit to a single exponential (Table 2). (C) Apparent single turnover kinetics for 3D^pol ^with Sym/Sub U with incorrect base U in the presence of cold trap. 1 μM 3D^pol ^and 1 μM Sym/Sub U were incubated with 750 (squares) or 1500 μM UTP (circles) for the indicated time (in minutes) as described in Methods, except that ten-fold molar excess unlabeled Sym/Sub U was added immediately after reaction initiation. Data (from all products) were plotted as a function of time in seconds and fit to a single exponential (Table 2).

To simulate single turnover conditions for incorrect base incorporation, we next used two different traps [21]. First, we employed a heparin trap. The cationic polysaccharide heparin rapidly inactivates polymerases by inhibiting re-initiation. To identify the minimum heparin concentration that inhibited re-initiation, 3D^pol ^(with 1 μM Sym/Sub U and 500 μM ATP) was incubated with increasing concentrations of heparin. Data were fit to a single exponential for inhibition (Fig. 4A). 100 nM was the lowest concentration found to inhibit 3D^pol^, similar to other work [20]. Therefore, incorrect incorporation reactions were performed as above except that 100 nM was added immediately after reaction initiation. Larger forms were reduced and yield was less than 500 nM (Fig. 4B). These data were then used to derive kinetic parameters (Table 2). Kinetic values derived by estimating single turnovers in untrapped reactions were not radically different than those derived by considering all products of heparin reactions.

To corroborate these results, incorrect incorporation reactions, identical to those described above, were also performed in the presence of 10-fold excess unlabeled Sym/Sub, added immediately after initiating the reaction. Any incorporation due to dissociation and re-initiation would be expected to be invisible in the radio-isotopic analysis. Apparent single turnover conditions were again achieved, as assessed by reduced generation of larger products and total product yield not significantly over 500 nM (Fig. 4C). These data were similarly used to derive amplitude and observed reaction rate; which were then used to derive *K*_*d *_and *k*_*pol*_. (Table 2). Including all products in calculating yield with the two trapping approaches (cold and heparin) yielded not extremely divergent kinetic values to untrapped experiments when apparent multiple turnover data were disregarded. Therefore, for the remaining analyses, untrapped reactions were performed, but product yield greater than 500 nM and products larger than 11 mers were disregarded for kinetic value derivations.

### 5. Extremely inefficient incorrect reactions

We proceeded to measure kinetic parameters for all possible mismatches (Table 3). However, the inefficient incorporation of incorrect bases had additional consequences during this analysis. For some reactions, so few data points were available that, although kinetic values were calculable by computer analysis, no standard error was obtained (SSU with 1500 μM UTP, SSC with 750 and 1500 μM CTP, SSA with 1500 μM GTP, SSA with 750 μM ATP, SSG with 750 μM ATP). Moreover, in some of these (SSC with 750 μM CTP, SSA with 1500 μM GTP, SSA with 750 μM ATP and SSG with 750 μM ATP), the data did not approximate a single exponential reaction. In these cases, "time zero" and the datapoint with highest incorporation were used to estimate amplitude and reaction rate. The combined derived values were used to estimate *K*_*d *_and *k*_*pol *_for incorrect base incorporation and then to calculate fidelity (Table 4). Moreover, amplitudes varied considerably, presumably due to enzyme inactivation during inefficient binding and catalysis. Therefore, fidelity values derived from incomplete data must be considered estimates only. However, at the present time, this appears to be the only experimental approach for assessing these extremely inefficient reactions.

**Table 3:** 

**Sym-Sub**	**NTP**	**[NTP] (μM)**	**Amplitude (nM)**	**k_**obs **_(sec-1)**
U	G^1,2^	375	350 (± 24)	1.0 (± 0.26) × 10^-3^
		750	200 (± 58)	1.5 (± 0.62) × 10^-3^
		1500	300 (± 24)	2.2 (± 0.54) × 10^-3^
	U^1,2^	750	640 (± 54)	3.5 (± 0.69) × 10^-4^
		1500	490 (± 44)	8.9 (± 0.29) × 10^-4^
		3000	380 (± 14)	2.8 (± 0.27) × 10^-3^
	C	1500	180 (± 35)	1.8 (± 0.64) × 10^-4^
		3000	76 (± 13)	2.7 (± 1.2) × 10^-4^
C	A	5	47 (± 14)	2.4 (± 1.1) × 10^-3^
		10	85 (± 16)	8.30 (± 0.86) × 10^-3^
	U^1,2^	100	34 (± 4.7)	2.30 (± 0.82) × 10^-3^
		200	79 (± 28)	2.80 (± 1.7) × 10^-3^
		750	82 (± 6.3)	6.50 (± 1.6) × 10^-3^
		1500	53 (± 7.2)	6.2 (± 2.0) × 10^-3^
	C^3^	750	470	9.3 × 10^-4^
		1500	640	1.2 × 10^-3^
A	C1	750	340 (± 26)	1.3 (± 0.21) × 10^-3^
		1500	250 (± 17)	5.1 (± 2.3) × 10^-3^
	G	750	270 (± 100)	1.8 (± 0.74) × 10^-4^
		1500	110	8.5 × 10^-4^
	A3	750	5	1.6 × 10^-3^
		1500	23 (± 5.2)	1.7 (± 1.0) × 10^-3^
G	U	750	190 (± 6.9)	1.3 (± 1.0) 10^-3^
		1500	280 (± 26)	8.3 (± 1.4) × 10^-4^
	A^1,3^	750	25	2.5 × 10^-5^
		1500	18	2.1 × 10^-5^
	G^3^	750	520	3.4 × 10^-5^
		1500	540	3.6 × 10^-5^

^1^Higher molecular weight products (12-, 13- and/or 14 mers) were observed, but data not included to estimate single turnovers.

^2^Data over 500 nM omitted.

^3^So few data points were available that there was no standard error for the single exponential calculations. In some cases, incorporation was so inefficient that data did not resemble a single exponential. Therefore, zero and the highest datapoint were used, yielding no standard error.

**Table 4:** Pre-steady state fidelity

**Sym-Sub**	**NTP**	***K*_*d *_(μM)**	***k*_*pol *_(sec-1)**	**k_*pol*_/K_*d *_(μM -1 sec-1)**	**Fidelity**^**1**^
U	A	120 ± 74	74 ± 22	0.62	-
	G^2^	1100 ± 100	0.004 (± 3.2) × 10^-4^	3.6 × 10^-6^	1.7 × 10^5^
	U	1.7 (± 0.53) × 10^5^	0.14 ± 0.053	8.2 × 10^-7^	7.5 × 10^5^
	C^3^	3000	5.4 × 10^-4^	1.8 × 10^-7^	3.4 × 10^6^
C	G	1.6 ± 2.3	15 ± 9.2	9.4	-
	A	1.0 × 10^4^	7.9	7.9 × 10^-4^	1.2 × 10^4^
	U	270 ± 120	0.0079 ± 0.0014	2.9 × 10^-5^	3.2 × 10^5^
	C^3^	620	0.0017	2.7 × 10^-6^	3.4 × 10^6^
A	U	27 ± 0.95	84 ± 0.48	3.1	-
	C	6.0 (± 0.70) × 10^5^	1.8 ± 0.55	3.0 × 10^-6^	1.0 × 10^6^
	G	1.0 (± 0.12) × 10^5^	0.051 ± 0.014	5.1 × 10^-7^	6.1 × 10^6^
	A	1.6 (± 0.032) × 10^5^	0.18 ± 0.0069	1.1 × 10^-6^	2.8 × 10^6^
G	C	44 ± 7.2	55 ± 3.8	1.3	-
	U^3^	230	0.002	8.7 × 10^-6^	1.4 × 10^5^
	A^3^	1.0 × 10^5^	0.015	1.5 × 10^-7^	8.3 × 10^6^
	G	1.0 (± 0.0071) × 10^5^	0.0029 (± 8.6) × 10^-4^	2.9 × 10^-8^	4.3 × 10^7^

^1^(*k*_*pol*_/*K*_*d*_)c + (*k*_*pol*_/*K*_*d*_)i/(*k*_*pol*_/*K*_*d*_)i. *k*_*pol *_and *K*_*d *_values from Table IV.

^2^The last two rows (for each sym/sub) represent transversion mutations.

^3^Kinetic values for which no error is reported were derived from only two NTP concentrations (see Table III).

### 6. Simulation

**Figure 5:**
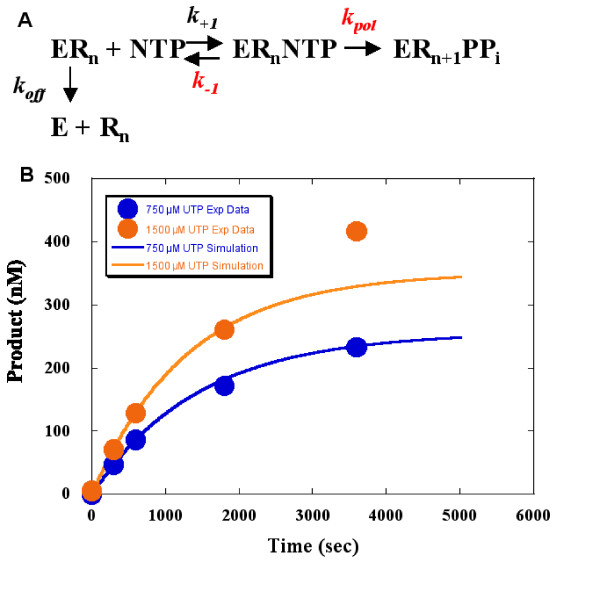
**Simulation of Sym/Sub U with UTP**. (A) Hypothesized mechanism used for simulation. E=Enzyme, R_*n*_=Template/Primer RNA, ER_*n*_=active complex. The nonvarying parameters were: ER_*n*_: 500 nM, NTPs: 750 or 1500 μM, *k*_+1 _: 10 μM^-1 ^sec^-1 ^and *k*_*off *_: 5 × 10^4 ^sec^-1^. In the simulation, which is based on the mechanism in [19]. *k*_*pol *_and *k*_-1 _(both in gray) were varied. (B) Data and simulated fit. Experimentally derived products formed with 750 (dark gray circles/blue) or 1500 μM (light gray circles/orange) UTP were plotted as a function of time (in seconds). Simulated fits (curves) were superimposed with same color scheme.

The detection of apparent multiple turnovers, greater than 500 nM product and extremely inefficient incorporation suggested that incorrect incorporation may sometimes be slower than enzyme RNA complex dissociation. Since 3D^pol ^is inactivated upon complex dissociation, this would cause a changing concentration of active enzyme, negating assumptions underlying the analysis. However, multiple simultaneous reactions (in this case, catalysis and enzyme inactivation) may be modeled, simulated and compared to experimental data. Experimental data from Sym/Sub U with 750 and 1500 μM UTP were fit to such a simulation (Fig. 5). In this simulation, *k*_+1_(10 μM^-1 ^sec^-1^) and *k*_*off *_(5 × 10^4 ^sec^-1^) [22], were not varied. *k*_*pol *_and *k*_-1 _could not be varied more than 5-fold. With the simulated values of 1 × 10^4 ^sec^-1 ^for k_-1 _{yielding 1000 μM for *K*_*d*_, (*k*_-1_/k_+1_)} and 1 × 10^-3 ^sec^-1 ^for *k*_*pol*_, the simulated curves fit the experimental data well. Also, the simulated *k*_*pol *_is in agreement with the derived value (Table 4). Therefore, the results of the simulation support our derived data. This fit supports the interpretation that two reactions are simultaneously occurring. Thus incorrect base catalysis is so inefficient that it occurs on the same time scale as dissociation and enzyme inactivation. This finding also helps explain some of the technical difficulties with measuring incorrect base incorporation.

## Discussion

In this work, we present the first pre-steady state fidelity measurements for poliovirus polymerase 3D^pol ^for all twelve possible mismatches. Most of these mismatch reactions are extremely inefficient. This inefficiency reflects an unexpected high fidelity. However, this inefficiency made some of the reactions, in particular transversions, almost unmeasurable. A number of technical difficulties, apparently inherent in this inefficiency, occasionally precluded a comprehensive, conventional pre-steady state analysis. Nevertheless, we developed procedures for deriving estimated kinetic values under these adverse experimental conditions. Future improved methods may determine the accuracy of these estimates.

We observed apparent greater than 500 nM product and unexpectedly long products; we interpreted this as multiple turnovers during ostensibly single turnover conditions. To confirm this, we used two traps to inhibit multiple turnovers. We found that kinetic values derived in the presence of traps were similar to values derived after disregarding data from apparent multiple turnovers. Moreover, computer simulations confirmed that, in the presence of incorrect NTP, the dissociation reaction interferes with catalysis. These phenomena made pre-steady state, single turnover reactions for inefficient incorporations challenging, since at least two reactions are occurring simultaneously. It seems clear that, in this case, multiple turnovers are secondary to the extreme inefficiency of incorrect incorporation. However, enzyme inactivation is apparently slower than re-association. Although some of our derived values must be considered tentative, the overall analysis presents a foundation upon which a fundamental understanding of viral quasi-species may be achieved.

We found that 3D^pol ^was inhibited by NTP concentrations higher than 2 mM. Polymerase inhibition by high nucleotide concentration has been noted previously: T7 DNA polymerase is inhibited by >4 mM dNTP, even in the presence of 12.5 mM MgCl_2 _[21]. Since some derived *K*_*d *_values were greater than 10^6 ^μM, this inhibition precluded experimentally approaching NTP *K*_*d *_values. It may be argued that the apparent multiple turnovers with incorrect base are due to artifactually slow reactions, secondary to concentrations of NTP significantly below *K*_*d*_. At the present time, this cannot be eliminated as an interpretation. However, this is not experimentally testable with current knowledge. Moreover, it is unlikely that under physiologically relevant conditions, NTP concentrations would approach such high levels, so the relevance of precisely determining such high *K*_*d *_values is minimal.

We performed a simulation to confirm that catalysis was so slow that a second reaction, enzyme RNA complex dissociation was interfering with the assumption of a single turnover. The results of the simulation supported this interpretation with two exceptions: the simulated *K*_*d *_is 4.3-fold higher than the experimentally derived value and one datapoint, the last point with 1500 μM UTP (Fig. 5) does not fit the simulated curve. These discrepancies may be due to an altered complex dissociation rate, not accounted for in the simulation. This alteration may be a direct or indirect result of the inefficient catalysis: these data do not address this issue. Future studies will be required to determine whether a mismatch incorporation alters enzyme RNA complex dissociation.

The fidelity values derived in this work are based on a minimal, nonphysiological, template/primer. It is possible that other sequences may yield different kinetic values. Since the exclusive polypeptide component of this *in vitro *reaction is polymerase, necessary future experiments will include additional viral factors, such as 2C, 3A, 3AB and 3CD, known to comprise the replication complex [23-29]. In addition, host factors may modulate the reaction [30]. Viral replication for this nonenveloped virus occurs on host membranes and requires active cellular lipid synthesis [31,32]. The enzymological roles of these factors in catalysis and fidelity remain to be determined. However, it is likely that the basic underlying finding, that 3D^pol ^has relatively high fidelity, will not differ appreciably. Moreover, the current analysis is extremely time- and labor-intensive; therefore, improved assay systems for RdRP fidelity may predate repeating similar analyses with alternative templates. Either way, measuring fidelity in the context of viral sequences and factors will be essential to understanding the biology of viral RdRPs.

The fidelity values derived here for PV 3D^pol ^are much higher than would be predicted by poliovirus sequence variation rates (while evolving in human populations), which is about 0.02 mutations per year [33]. However, this value is an average of all mutations. An analysis of base-specific mutations is required to compare the values derived here to virological sequence variation rates. Other RdRPs have not been similarly analyzed. However, HIV RT, an RdDP, displays fidelity of 10^3 ^– 10^4^, values more consistent with overall HIV sequence variation [18].

Absence of exonucleolytic editing function in RdRPs is often cited as the reason for high sequence variation (quasi-species) of RNA viruses [1]. However, absence of an exonucleolytic editing activity does not account for high sequence variation of poliovirus, since the values measured here are much less error-prone than predicted. Therefore, systematic studies to determine additional contributions to RNA sequence variation are required. Candidate variables include: viral or host modulating factors, recombination, deletion, untemplated insertions, base tautomerization, RNA editing and selection. In particular, the apparent dissociation of enzyme RNA complex in the presence of incorrect base may suggest that recombination is induced by mismatches. 3D^pol ^has a proclivity towards template switching [20]. Future studies must be directed towards understanding virological mechanisms that modulate polymerase fidelity in producing highly variable quasi-species.

These results also suggest that mechanisms mediating the anti-viral activity of lethal mutagenesis may be more complex than originally proposed. Since many RNA viruses have been shown to be susceptible [10], a structural and mechanistic understanding of RNA polymerase fidelity may permit rational drug design or high throughput pharmaceutical screening of improved lethal mutagenic agents. A new generation of novel mutagens with increased specificity for viral polymerases are being explored [34]. Another possibility is that, although existing lethal mutagenesis strategies target increasing mutation, our work may reveal the usefulness of increasing fidelity. This could render viruses less prone to drug resistance. In this innovative approach, novel "fidelity" drugs could be used in combination with existing anti-virals to enhance their efficacy. It is possible that our findings will be generalizable to other RdRPs. Future studies of RdRP fidelity will be required to resolve these issues.

## Conclusion

RdRP incorporation of incorrect nucleosides is unexpectedly inefficient, making precise determination of kinetic parameters experimentally challenging.

We found that fidelity for poliovirus polymerase 3D^pol ^ranges from 1.2 × 10^4 ^to 1.0 × 10^6 ^for transition mutations and 3.2 × 10^5 ^to 4.3 × 10^7 ^for transversion mutations.

Viral or host factors, or template-switch recombination may contribute to high virus sequence variation.

## Methods

### Poliovirus polymerase 3D^pol ^purification

Recombinant PV polymerase (3D^pol^) in pET26Ub-3D (obtained from C. Cameron) was purified as described [35], with minor modifications. 3D^pol ^is initially produced as a C-terminal fusion to *Saccaromyces cerevisiae *ubiquitin. The host strain [BL21 (DE3) pCG1] expresses Ubp1, an ubiquitin-specific C-terminal protease, permitting production of a stable authentic glycine N-terminus of 3D^pol^, after IPTG induction. Small aliquots and bulk pellets from 750 ml induction were frozen prior to processing. Activity was confirmed prior to purification. The ammonium sulfate pellet was resuspended in 20 ml dialysis buffer consisting of: 25 mM HEPES pH 7.5, 50 mM NaCl, 0.02% NaN_3 _and 0.1 mM EDTA plus fresh 2 mM DTT, 1 mM PMSF and 2.8 ug/ml pepstatin A. After overnight dialysis at 4°C, the dialysate was centrifuged at 17.7 Krpm for 30 min, 4°C, in a JA-20 rotor, Beckman J2-21 centrifuge. Glycerol (to 15%) was added to the supernatant, which was then filtered sequentially through 0.45 and 0.22 micron syringe filters. After adding fresh protease inhibitors, the material was chromatographed on a 20 ml Pharmacia Sepharose SPHP XK16/20 ("S20") column using a Bio-Rad Biologic Duo-Flow instrument. This and subsequent columns were loaded and rinsed (3 column volumes) in 0.01 M NaCl, 25 μM HEPES pH 8.5, 0.02% NaN_3_, 0.1 mM EDTA, 15% glycerol, 0.5% BOG, and 2 mM DTT. Proteins were eluted in 0.005 to 0.35 M NaCl gradient. The protein peak usually eluted at 0.23–0.24 M NaCl. Peak fractions were pooled, filtered through a 0.22 μM filter and adjusted to 0.01 M NaCl in the same buffer except that Tris was substituted for HEPES. Pooled fractions were chromatographed on Q20 column, with a gradient of 0.005 to 0.5 M NaCl. Peak fractions (about 0.27 M NaCl) were pooled, and activity assessed. Several preparations were used in the experiments reported here, with comparable experimental results. The first preparation consisted of S20, followed by Q20. Subsequent preparations consisted of sequential S20, Q5 and UNO columns. The concentrating UNO column (Q 1.3 ml) was eluted in two steps of 0.5 M NaCl. If storage was required between purification steps, all fractions were stored at -80°C. After the final column, 10–25 ul analytical samples were stored at -80°C. Care was taken to ensure that final glycerol concentration was below 3% during experiments. Optical density at 280 nm was measured and protein concentration calculated, using MW of 53,000 Da and extinction co-efficient of 720,000 μM ^-1 ^cm^-1^. During pilot studies, fractions were assessed by activity, Coommassie-stained PAGE and western blot analyses (monoclonal antibody from J. Lyle and K. Kirkegaard [Stanford]) after each column. After establishing reproducible purification conditions, the procedures were streamlined, by minimizing intermediate assessments. Typically, a 750 ml culture yielded about 5 ml of 20–70 μM enzyme, after active site titration.

Each preparation was characterized in a rapid burst experiment for determination of the number of active sites (below) prior to experimental use.

Activity during purification was assessed in a poly (rA)/U incorporation assay consisting of enzyme, template (0.15 μM poly(rA)_400_, primer (1.8 μM dT_15_), 500 μM UTP and 0.4 μCi/ul α-[^32^P]UTP in buffer containing 50 mM HEPES pH7.5, 60 μM ZnCl_2_, 10 mM β-mercaptoethanol, and 5 mM MgCl_2 _[15]. Samples were incubated 30 min at 30°C. Duplicate aliquots were quantified by liquid scintillation after removal of unincorporated nucleotides, which was accomplished by rinsing spotted GF/A glass filter discs in 250 ml dibasic sodium phosphate three times (10 min each), 100% cold EtOH and drying. After subtracting background ("no template") samples, active lysates generally incorporated several hundred-fold more isotope than "time zero" samples.

### RNA substrate preparation

To measure polymerase fidelity *in vitro*, we used a synthetic self-annealing 10-mer RNA oligonucleotide (Dharmacon) called "Symmetrical Substrate" ("Sym/Sub" or "SS") designed by Dr. Cameron for analysis of 3D^pol ^(Fig. 1). Sym/Subs are designated by their first template base; for example, GCAUGGGCCC is called Sym/SubU or SSU. RNAs were deprotected, desalted and gel-purified by Dharmacon. Sym/Sub RNA was 5'-end-labeled with γ-[^32^P-ATP] using polynucleotide kinase (New England BioLabs) following the manufacturer's instructions. Unincorporated ATP was removed using a spin column (Sephadex G-25, Sigma). Labeled and unlabeled Sym/Sub were mixed in a ratio of approximately 1:100, melted for 1 min at 90°C and permitted to self-anneal by cooling to 10°C at 6° per min, using an MJ Research thermocycler. The annealing reaction was assessed by comparing native and denatured RNA mobility in a native 20% polyacrylamide gel.

### Pre-steady state analysis

For each experiment, active complexes of enzyme and annealed Sym/Sub were pre-formed by a 90 second incubation at room temperature, as required for 3D^pol ^Sym/Sub assembly and kept on ice until used [15]. Single turnover control reactions, rapid burst active site titrations and pre-steady state reactions with correct NTP were performed using rapid quench instrumentation in a Kintek, Inc Rapid Quench instrument. For these experiments, two-fold concentrated half-reactions were assembled such that addition of NTP initiated the biochemical reaction. Reactions were quenched with EDTA to a final concentration of 0.3 M.

To determine the number of active sites (after purification but before kinetic measurements), rapid burst experiments in which Sym/Sub was varied from 1 to 12-fold excess over enzyme, were performed. Product formation at the amplitude plateau was used to calculate active sites. Enzyme concentration was adjusted to reflect moles of active sites.

After determination of number of active sites, a series of enzyme calibration experiments to ensure single turnover conditions were performed. Initial NTP concentration was high (500 μM for SSU with ATP) to ensure that it was not limiting. Enzyme was varied 1–4-fold over substrate. Single turnover conditions were confirmed when reaction rate did not increase with increased enzyme. Under these conditions, amplitude did not significantly vary. For pre-steady state reactions, Sym/Sub concentration was 1 μM (= 0.5 μM duplex). When using Sym/Sub U and Sym/Sub C, final enzyme concentration was 1 μM while for Sym/Sub G and Sym/Sub A, final enzyme concentration was 2.5 and 2.8 μM, respectively, based on single turnover control reactions. Other reaction constituents were: 60 μM ZnCl_2_, 50 mM HEPES pH 7.6 and 5 mM MgCl_2_. Input and product(s) were electrophoretically separated in 20% acrylamide, 1.7% bis-acrylamide, 7 M Urea, 1 × TBE PAGE at 75 Watts, maintained at 55°C. Conditions for pre-steady state analysis of incorrect NTPs were performed and analyzed identically, except that they were not performed with rapid quench instrumentation, but on the benchtop with manual additions.

### Data analysis

20% gels were visualized on a Bio-Rad Personal Molecular Imager FX Phospho-Image analyzer and quantified using Quan-One software. Gel background was subtracted from each datapoint. Product yield in nM was determined by first dividing product by total (product [s] plus remaining input Sym/Sub). Input Sym/Sub was 1000 nM, but because only 0.5 of end-labeled RNA will be used in a single turnover (2 labels per duplex), the product ratio was then multiplied by 500 nM. Then, the zero timepoint product was subtract from all values. These yield values were plotted as a function of time. In most cases where more than one product was formed, only 11 mers were used to determine nM product, although all products were used to derive "total" values. Exceptions to this are discussed in the text and figure legends. For correct base incorporation with Sym/Sub C and Sym/Sub A, (for unknown reasons) plateau values were not the same, so data were normalized to the same endpoint, by multiplying data from the lower set by the same factor. Normalizing did not change observed reaction rates. Data were fit to a first order process with Sigmaplot generating Amplitude and *k*_*obsd *_for each reaction using a single exponential equation. *k*_*obsd *_values were then plotted as a function of NTP concentration. These data were fit to a hyperbola, to derive *K*_*d *_and *k*_*pol*_. The formula: {(*k*_*pol*_/*K*_*d*_)c + (*k*_*pol*_/*K*_*d*_)i}/(*k*_*pol*_/*K*_*d*_)i, where "c" is correct nucleotide and "i" is incorrect nucleotide, was used to calculate fidelity [18]. Two significant digits were used throughout. Error was derived by the computer program Sigmaplot.

The simulation (shown in Fig. 5) was performed by J. Arnold (Pennsylvania State University) with the program KinTekSim [36]. When performing the simulations, first the fixedparameters are input. These included: concentrations of 3D^pol ^Sym/SubU complex (500 nM), nucleotide (750 and 1500 μM), the rate of dissociation of the 3D^pol ^Sym/Sub U complex [*k*_*off*_: 0.0005 s^-1 ^[15]] and the on-rate for nucleotide binding (*k*_+1_: 10 μM^-1 ^sec^-1^). Reasonable values for *k*_*pol *_(based upon the rate obtained from fitting experimental data from the maximum concentration of nucleotide, in this case 1500 μM, to a single exponential) and *k*_-1 _are entered. Each value was adjusted to obtain best fit of the data. When obtaining simulations, there is only one good solution to obtain the best fit to the experimental data. Neither *k*_-1 _or *k*_*pol *_could be varied individually by more than 5-fold and still obtain a good fit. The kinetic values used in the simulation were: *k*_+1 _= 10 μM s^-1^, *k*_-1 _= 1 × 10^4 ^s^-1^, *K*_*d *_= *k*_+1_/*k*_-1 _= 1000 μM, 1 s *k*_*pol *_= 10^-3 ^s^-1^, *k*_*off *_= 5 × 10^-4 ^s^-1^.

## Abbreviations

HIV = human immunodeficiency virus

PV = poliovirus

BOG = n-Octyl-beta-D-glucopyranoside

RdDPs = RNA-dependent DNA polymerases

RdRPs = RNA-dependent RNA polymerases

Sym/Sub = Symmetrical Substrate.

SS = Symmetrical Substrate.

RT = Reverse Transcriptase.

## Competing interests

The author(s) declare that they have no completing interests.

## Authors' contributions

MF conceived and designed the study, performed data analysis and wrote the manuscript. KE performed protein purification, characterization and all kinetic studies. JV participated in the design of the study and provided necessary expertise in polymerase enzyme kinetics as well as unlimited access to the sole source of the relevant instrumentation.
